# Supplementary material for: Quantification of Furosine (Nε-(2-Furoylmethyl)-l-lysine) in Different Parts of Velvet Antler with Various Processing Methods and Factors Affecting Its Formation
Source: Molecules. 2019 Mar 31;24(7):1255. doi: 10.3390/molecules24071255 (PMC6479359; doi:10.3390/molecules24071255)
Supplement: Supplementary file 1 [file molecules-24-01255-s001.pdf]

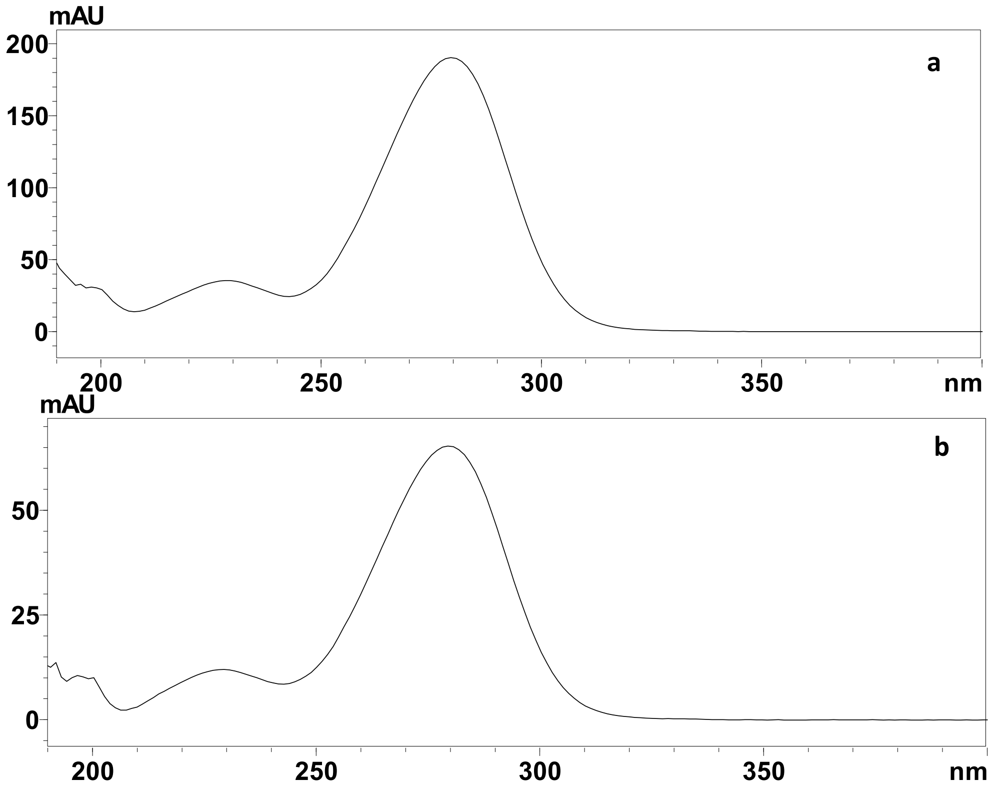

Figure. 1. UV determination of furosine standards (a) and powder slices of boiled velvet antler samples (b).

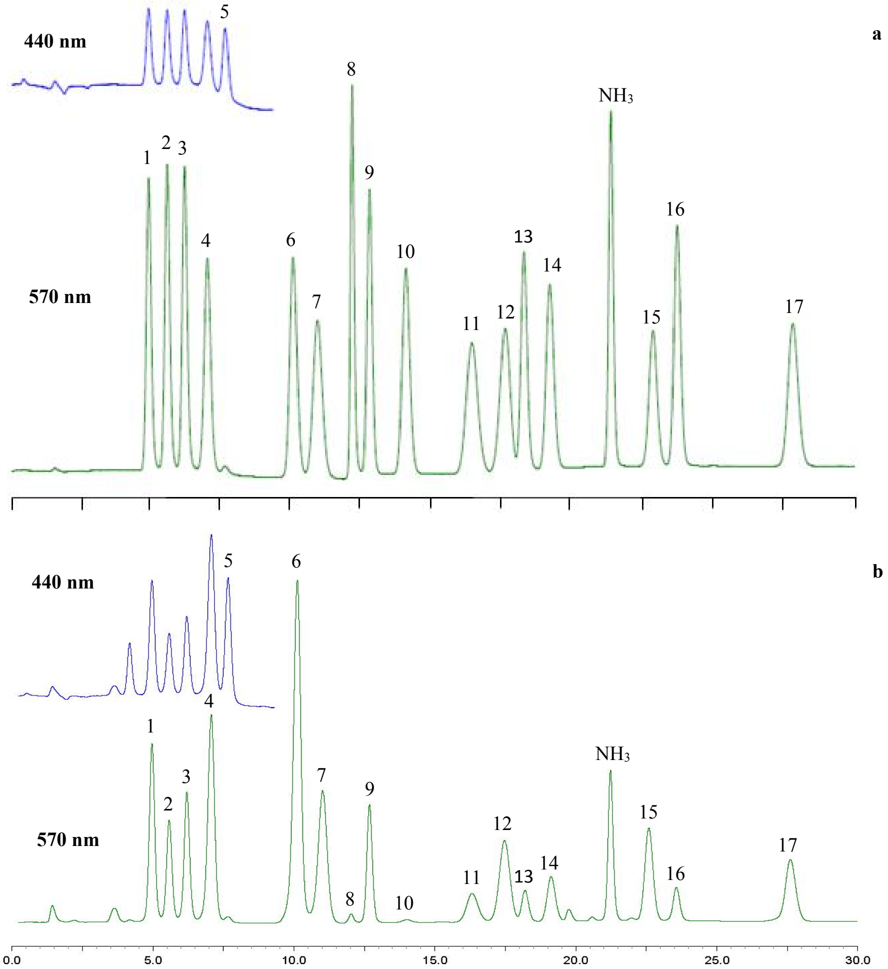

Figure.2. Chormatograms of the seventeen amino acids in (a) mixed standard and (b) powder slices of freeze-drying antler velvet. Peak numbers 1~17 represent aspartic acid, threonine, serine, glutamic acid, proline, glycine, alanine, cysteine, valine, methionine, isoleucine, leucine, tyrosine, phenylalanine, lysine, histidine, argnine.

Table 1 Seventeen amino acids contents in processed antler velvet determined by an automatic amino acid analyzer.

Samples		Contents /g/100g								
		**Asp**	**Thr**	**Ser**	**Glu**	**Gly**	**Ala**	**Cys**	**Val**	**Met**
Freezee-dried	W	7.29±0.49	3.51±0.17	3.85±0.11	10.90±0.44	11.96±0.61	7.18±0.48	0.58±0.02	4.61±0.11	0.32±0.01
	P	5.30±0.10	2.49±0.07	2.65±0.04	7.53±0.59	8.80±0.58	5.24±0.08	0.28±0.01	3.39±0.01	0.19±0.01
	G	4.88±0.07	2.24±0.08	2.52±0.02	7.22±0.22	10.01±0.31	5.43±0.06	0.32±0.02	3.00±0.04	0.12±0.01
	B	4.30±0.12	1.92±0.09	2.23±0.18	6.68±0.22	10.79±0.25	5.40±0.17	0.18±0.04	2.57±0.12	0.04±0.02
	E	5.15±0.21	2.33±0.15	2.65±0.23	7.79±0.31	10.99±0.26	5.79±0.09	0.33±0.03	3.11±0.14	0.18±0.01
Boiled	W	7.13±0.31	3.47±0.12	3.82±0.15	10.94±0.93	11.87±0.45	6.96±0.33	0.71±0.02	4.56±0.05	0.16±0.02
	P	5.15±0.04	2.42±0.04	2.64±0.05	7.75±0.42	9.49±0.15	5.47±0.22	0.36±0.02	3.24±0.04	0.15±0.01
	G	4.76±0.03	2.15±0.06	2.42±0.01	7.08±0.23	10.19±0.05	5.56±0.06	0.23±0.00	2.95±0.06	0.06±0.01
	B	4.10±0.05	1.84±0.12	2.13±0.14	6.37±0.21	10.43±0.14	5.28±0.14	0.19±0.08	2.43±0.11	0.05±0.01
	E	5.22±0.11	2.40±0.08	2.76±0.12	8.00±0.22	11.50±0.23	6.09±0.31	0.20±0.07	3.16±0.09	0.05±0.02

Samples		Contents /g/100g								
								17		
		**Ile**	**Leu**	**Tyr**	**Phe**	**Lys**	**His**	**Arg**	**Pro**	**Total（TAA）**
Freezee-dried	W	2.64±0.10	6.64±0.13	2.30±0.01	3.65±0.09	5.87±0.20	2.04±0.04	6.69±0.41	8.61±0.54	88.64±3.30
	P	1.65±0.02	4.86±0.09	1.28±0.01	2.62±0.04	3.96±0.11	1.68±0.03	4.52±0.21	6.07±0.22	62.51±1.84
	G	1.49±0.05	4.20±0.05	1.21±0.01	2.37±0.02	3.76±0.03	1.39±0.02	4.76±0.07	6.83±0.09	61.75±0.90
	B	1.25±0.12	3.50±0.13	1.01±0.17	2.01±0.15	3.40±0.21	1.10±0.17	4.63±0.45	7.15±0.52	58.20±1.17
	E	1.70±0.09	4.35±0.24	1.28±0.09	2.42±0.14	4.02±0.13	1.37±0.07	5.12±0.34	7.42±0.49	68.00±1.23
Boiled	W	2.98±0.02	6.49±0.24	2.14±0.03	3.52±0.04	5.67±0.22	1.89±0.02	6.87±0.36	8.30±0.34	87.48±1.78
	P	1.72±0.05	4.59±0.13	1.23±0.01	2.53±0.04	3.92±0.14	1.49±0.07	4.65±0.10	6.49±0.12	62.29±0.82
	G	1.39±0.01	4.09±0.03	1.13±0.00	2.32±0.02	3.69±0.10	1.37±0.02	4.62±0.12	6.96±0.06	60.97±0.28
	B	1.17±0.12	3.28±0.11	0.91±0.04	1.93±0.11	3.15±0.12	1.04±0.11	4.33±0.35	6.99±0.17	55..62±1.74
	E	1.70±0.09	4.35±0.05	1.30±0.02	2.44±0.09	4.09±0.29	1.37±0.09	5.20±0.42	7.76±0.21	67.59±1.82

Samples		Contents /g/100g								
		**Asp**	**Thr**	**Ser**	**Glu**	**Gly**	**Ala**	**Cys**	**Val**	**Met**
Without blood	W	6.82±0.32	3.28±0.12	3.67±0.11	10.72±0.63	12.57±0.35	7.07±0.13	0.60±0.01	4.38±0.03	0.10±0.01
	P	5.20±0.21	2.44±0.02	2.73±0.05	8.35±0.26	10.62±0.21	5.67±0.11	0.45±0.01	3.18±0.07	0.22±0.00
	G	4.71±0.07	2.14±0.01	2.46±0.01	7.50±0.13	10.31±0.41	5.80±0.21	0.39±0.01	2.81±0.02	0.06±0.01
	B	3.87±0.21	1.77±0.11	2.05±0.12	6.34±0.25	9.45±0.33	4.72±0.27	0.30±0.18	2.27±0.09	0.05±0.01
	E	4.47±0.19	1.98±0.04	2.35±0.09	7.25±0.34	12.46±0.41	6.01±0.32	0.32±0.03	2.60±0.11	0.06±0.02
With blood	W	7.16±0.11	3.43±0.07	3.84±0.03	11.11±0.36	13.59±0.21	7.54±0.14	0.68±0.01	4.46±0.08	0.09±0.01
	P	5.88±0.12	2.80±0.02	2.99±0.03	8.52±0.12	11.19±0.10	6.54±0.05	0.48±0.01	3.84±0.05	0.18±0.01
	G	5.47±0.07	2.62±0.05	2.81±0.01	8.11±0.09	9.49±0.30	5.73±0.03	0.37±0.01	3.53±0.05	0.25±0.00
	B	4.36±0.19	1.95±0.11	2.29±0.08	6.95±0.15	11.84±0.21	5.85±0.07	0.11±0.05	2.61±0.11	0.04±0.02

E	5.04±0.18	2.33±0.13	2.46±0.10	7.40±0.14	10.37±0.14	5.88±0.05	0.33±0.03	3.25±0.18	0.12±0.00

**Samples**		**Contents /g/100g**								
		**Ile**	**Leu**	**Tyr**	**Phe**	**Lys**	**His**	**Arg**	**Pro**	**Total（TAA）**
Without blood	W	2.96±0.01	6.11±0.17	2.14±0.00	3.35±0.02	5.56±0.11	1.74±0.02	6.89±0.21	8.78±0.21	86.74±0.18
	P	1.99±0.03	4.49±0.11	1.35±0.00	2.48±0.04	4.01±0.09	1.27±0.03	5.01±0.11	7.19±0.14	66.65±0.74
	G	1.53±0.03	3.81±0.07	1.11±0.01	2.18±0.01	3.76±0.11	1.13±0.01	5.00±0.07	7.65±0.01	62.35±0.48
	B	1.26±0.11	3.15±0.14	0.89±0.14	1.74±0.14	3.01±0.12	0.88±0.11	4.11±0.21	6.28±0.15	52.34±0.72
	E	1.42±0.05	3.54±0.12	1.05±0.03	2.11±0.09	3.58±0.15	1.05±0.05	5.09±0.13	8.21±0.19	63.55±0.82
With blood	W	2.96±0.03	6.30±0.14	2.11±0.02	3.42±0.07	5.66±0.12	1.75±0.01	7.28±0.12	9.44±0.11	91.02±0.46
	P	1.77±0.04	5.33±0.07	1.57±0.02	3.01±0.02	4.81±0.02	1.83±0.01	5.42±0.05	7.90±0.09	74.06±0.34
	G	1.78±0.04	5.06±0.03	1.44±0.05	2.81±0.02	4.26±0.07	1.75±0.03	4.78±0.13	6.48±0.05	66.94±0.49
	B	1.31±0.08	3.52±0.11	1.02±0.14	2.04±0.09	3.48±0.15	1.05±0.05	4.88±0.15	7.89±0.18	61.06±0.46
	E	1.48±0.06	4.50±0.13	1.24±0.22	2.52±0.12	4.03±0.17	1.53±0.07	4.74±0.17	7.09±0.19	64.31±0.56

Code W, P, G, B and E indicates wax slices, powder slices, gauze slices, and bone slices, respectively.
